# MiR-124-3p Suppresses Glioma Cells Progression by Targeting STAT3/NAMPT and Inhibiting AKT/ERK Signaling

**DOI:** 10.2174/0115680096444128260128052644

**Published:** 2026-04-02

**Authors:** Yan Liu, Wei Zhao, Zhi Tang, Wenwu Liu, Lei Wang, Te Wang

**Affiliations:** 1 Department of Neurology, The Affiliated Changsha Central Hospital, Hengyang Medical School, University of South China, No. 161 Shaoshan road, Yuhua District, Changsha, 410004, Hunan, China;; 2 Department of Neurosurgery, Hunan Cancer Hospital and the Affiliated Cancer Hospital of Xiangya School of Medicine, Central South University. No. 283 Tongzipo Road, Yuelu District, Changsha, 410006, Hunan, China;; 3 Hunan Provincial University Key Laboratory of the Fundamental and Clinical Research on Multimodal Oncology, Changsha Medical University, No.1501 Leifeng Road, Wangcheng District, Changsha 410219, Hunan, China

**Keywords:** miR-124-3p, NAMPT, STAT3, AKT, ERK, glioma, tumor suppression, phosphorylation

## Abstract

**Background:**

In our preliminary study, bioinformatics analyses identified differentially expressed miRNAs and mRNAs in glioma versus peritumoral samples, with functional validation of multiple miRNA-mRNA regulatory axes. MicroRNA-124-3p (miR-124-3p)/nicotinamide phosphoribosyltransferase (NAMPT) was among these axes and may critically regulate glioma malignant phenotypes, and this study aimed to validate their regulatory interactions.

**Methods:**

We analyzed NAMPT expression in glioma tissues using clinical datasets from the Chinese Glioma Genome Atlas (CGGA). Functional effects of miR-124-3p and NAMPT were evaluated in U87 and U251 glioma cells using quantitative real-time PCR (qRT-PCR), Western blotting, colony formation assays, CCK-8 cell viability assays, Transwell invasion assays, and flow cytometry. The direct interaction between miR-124-3p and NAMPT was investigated using dual-luciferase reporter assays.

**Results:**

Clinical data revealed that elevated NAMPT expression correlated with poor prognosis, advanced pathological grade, and IDH-wild-type gliomas. MiR-124-3p overexpression significantly inhibited glioma cell proliferation, migration, and invasion while promoting apoptosis. Mechanistically, miR-124-3p downregulated NAMPT expression through regulation, leading to suppression of the p-AKT and p-ERK signaling pathways. Notably, NAMPT overexpression rescued the tumor-suppressive effects of miR-124-3p, confirming its critical role in this axis. However, dual-luciferase reporter assays ruled out a direct interaction between miR-124-3p and the NAMPT transcript, suggesting an indirect regulatory mechanism. Further investigation through WB and rescue experiments identified the transcription factor STAT3 as the key intermediary, serving as a functional bridge linking miR-124-3p to the regulation of NAMPT expression.

**Discussion:**

Accumulating evidence demonstrates that miR-124-3p functions as a potent tumor suppressor across diverse malignancies through distinct molecular mechanisms. STAT3, which is directly targeted by miR-124-3p, serves as the functional bridge linking miR-124-3p to NAMPT regulation. NAMPT, as a critical node in the NAD⁺ biosynthetic pathway, influences the malignant biological behavior of glioma through downstream PI3K/AKT and MEK/ERK signaling transduction pathways.

**Conclusion:**

MiR-124-3p functions as a tumor suppressor in glioma by repressing STAT3/NAMPT-mediated AKT/ERK signaling, highlighting its potential as a diagnostic biomarker and therapeutic target for glioma management.

## 1 INTRODUCTION

MicroRNAs(miRNAs), a class of endogenous non-coding RNAs typically 18-25 nucleotides in length, have emerged as highly promising biomarkers for tumor diagnosis and therapeutic targets [1, 2]. Their functional significance stems from their unique capacity to modulate gene expression at the post-transcriptional level through sequence-specific base pairing with target messenger RNAs (mRNAs), thereby regulating mRNA stability and/or translation efficiency. Accumulating evidence underscores the critical role of miRNAs in governing diverse physiological processes of glioma cells, including proliferation, differentiation, apoptosis, and metastasis [3-4]. Notably, dysregulated miRNA expression profiles and their complex cross-talk with corresponding target mRNAs have been increasingly recognized as valuable diagnostic and prognostic indicators across all glioma grades [5, 6].

In our preliminary studies, we leveraged bioinformatics analyses to compare glioma specimens with adjacent peritumoral tissues, successfully identifying 59 differentially expressed miRNAs and 419 differentially expressed mRNAs [7]. Subsequent experimental validation was performed to characterize the regulatory interactions between these miRNAs and mRNAs in glioma cell models. In subsequent studies, we systematically validated the regulatory roles of key miRNA-mRNA axes, including the miR-139-5p/GABRA1 axis, miRNA-338-3p/MYT1L axis, miR-383-5p/VEGFA axis, miR-138-5p/WEE1 axis, and miR-128-3p/KLHDC8A axis, in the malignant biological behaviors of glioma [8-11].

Previously, our studies identified that miRNAs primarily function as tumor suppressors in glioma, exerting inhibitory regulation over downstream target gene expression to modulate glioma cell behaviors such as proliferation and invasion. However, they employ distinct regulatory mechanisms. For instance, miR-139-5p regulates glioma progression via modulating GABRA1 expression, potentially linking to synaptic electrical activity, miR-383-5p acts through direct targeting of VEGFA, closely associating with the mTOR signaling pathway, and miR-338-3p influences the malignant biological behaviors of glioma by targeting MYT1L, which may be involved in cell cycle regulation [7-11].

Among the previously identified miRNA-mRNA candidate pairs, the regulatory axis involving miR-124-3p and NAMPT particularly captured our interest. A systematic review incorporating 1,120 initial results from 8 independent studies revealed that patients with low miR-124-3p expression exhibited a significantly higher Hazard Ratio (HR) for adverse outcomes compared to those with high miR-124-3p expression (*p* < 0.05) [12]. Furthermore, miR-124-3p is extractable from serum exosomes and may serve as a promising molecular biomarker for complementing clinical assessments of early tumor progression in patients with High-Grade Gliomas (HGG) undergoing post-surgical treatment [13]. Our preliminary bioinformatics analysis revealed a regulatory relationship between miR-124-3p and NAMPT, a critical node in the NAD⁺ biosynthetic pathway, and identified targeting this pathway as a promising therapeutic strategy for isocitrate dehydrogenase(IDH)-mutant gliomas [14]. To date, however, the functional interplay between miR-124-3p and NAMPT remains unexplored, motivating our investigation of their combined role in gliomas and the mechanisms underlying this interaction.

Energy metabolism plays a pivotal role in the malignant biological behavior of gliomas and represents a promising therapeutic target. Recent clinical trials have demonstrated that Vorasidenib significantly prolongs progression-free survival in patients with IDH1- or IDH2-mutant low-grade gliomas [15-17]. NAMPT functions as a rate-limiting enzyme in the NAD (nicotinamide adenine dinucleotide) biosynthetic salvage pathway [18]. NAD, a pivotal coenzyme, is central to cellular redox homeostasis and serves as an essential substrate for NAD-dependent enzymes. Beyond its role in redox balance, NAMPT exerts pleiotropic effects on key cellular processes, including cell cycle progression, apoptosis, DNA repair, circadian rhythm regulation, chromatin dynamics, telomerase activity, intracellular calcium mobilization, and transcriptional control [19]. Based on these observations, we hypothesize that miR-124-3p may regulate glioma progression by modulating NAMPT expression, thereby influencing glioma cell metabolism and potentially improving patient prognosis.

## 2 METHODS

### 2.1 Cell Culture and Transfection

Human Glioblastoma Multiforme (GBM) cell lines U87 and U251 were obtained from BeNa Culture Collection (BNCC, China) and maintained in Dulbecco's Modified Eagle Medium (DMEM) supplemented with 10% Fetal Bovine Serum (FBS; Gibco, USA) at 37°C in a humidified incubator with 5% CO₂. For transfections, cells were seeded in 6-well plates at 70–80% confluency and transiently transfected with miR-124-3p mimic (50 nM), NAMPT-overexpressing plasmid (pcDNA3.1-NAMPT; 2μg), or corresponding negative controls (Honorgene, Hunan, China) using Lipofectamine 2000 (Invitrogen, Carlsbad, CA, USA) according to the manufacturer’s protocol. Transfection efficiency was verified *via* Quantitative Real-Time Polymerase Chain Reaction (qRT-PCR) 48 hours post-transfection.

### 2.2 Quantitative Real-Time PCR (qRT-PCR)

Total RNA was extracted from transfected cells using TRIzol reagent (Thermo Fisher Scientific, USA) following the manufacturer’s instructions. Complementary DNA (cDNA) synthesis was performed using a SuperRT RT kit (CWBio, China) with 200 ng of total RNA. The qRT-PCR was conducted on a CFX Connect Real-Time PCR Detection System (Bio-Rad, USA) using SYBR Green PCR Master Mix (CWBio, China). The thermal cycling conditions included initial denaturation at 95°C for 10 minutes, followed by 40 cycles of 95°C for 15 seconds and 60°C for 30 seconds. Relative gene expression was calculated using the 2⁻ΔΔCt method and normalized to β-actin. Primer sequences are listed in Table **1**. All experiments were performed in triplicate.

### 2.3 Western Blot Analysis

Protein lysates were prepared using RIPA buffer (Beyotime, China) supplemented with protease/phosphatase inhibitors. Protein concentration was quantified using the BCA assay (Thermo Fisher Scientific). Equal amounts of protein (20 μg) were resolved on 10% sodium dodecyl sulfate-polyacrylamide gels (SDS-PAGE) and transferred to polyvinylidene fluoride (PVDF) membranes (Millipore, USA). Membranes were blocked with 5% non-fat milk in Tris-buffered saline containing 0.1% Tween-20 (TBST) for 1 hour at room temperature, followed by overnight incubation at 4°C with primary antibodies, such as, NAMPT(1:2000, Abiowell, China), ERK(1:2000, Abiowell, China), p-ERK (Thr202 / T185, 1:2000, Abiowell, China), AKT(1:2000, Abiowell, China), p-AKT(Ser473, 1:2000, Abiowell, China), STAT3 (1:500, Abiowell, China), p-STAT3 (Tyr705, 1:1000, Abiowell, China), Bax (1:1000, Abiowell, China), Bcl-2 (1:500, Abiowell, China) and β-actin (1:5000, Proteintech, USA). After washing with TBST, membranes were incubated with HRP-conjugated secondary antibodies (anti-mouse and anti-rabbit, 1:5000, Abiowell, China) for 1 hour at room temperature. Protein bands were visualized using enhanced chemiluminescence (ECL; Millipore) and quantified via densitometry (Image J software).

### 2.4 Cell Viability Assay

Cellular proliferation was evaluated using the Cell Counting Kit-8 (CCK-8; Tongren, Japan). At 6 hours post-transfection, U87 and U251 cells were seeded into 24-well plates and maintained in 300 μL of complete cell culture medium. Following a 48-hour incubation period, 30 μL of CCK-8 reagent was added to each well, and the plates were further incubated at 37°C for 4 hours. The Optical Density (OD) at 450 nm was measured using a microplate reader (BioTek; Huisong, Shenzhen, China).

### 2.5 Colony Formation Assay

Six hours after transfection, U87 and U251 cells were plated in 6-well plates (Nunc, Roskilde, Denmark) with a seeding density of 200 cells per well. After a 14-day incubation period, formed cell colonies were fixed using alcohol, stained with 0.3% crystal violet solution (Solarbio, Beijing, China) for 30 minutes, and subsequently rinsed twice with deionized water.

### 2.6 Apoptosis Assay

Cell apoptosis rates across distinct treatment groups were assessed using the Apoptosis Detection Kit (#KGA1030, KeyGen BioTECH, China). Cells underwent three consecutive washes with PBS (Hyclone, USA) followed by centrifugation. The pellet was then resuspended in 500 μL of binding buffer, centrifuged again at 2000 rpm for 5 minutes, and subsequently incubated with 500 μL of binding buffer, 5 μL of Annexin V-APC, and 5 μL of propidium iodide.

### 2.7 Transwell Migration/Invasion Assay

To evaluate the migratory and invasive capabilities of U87 and U251 cells, Transwell assays were performed 6 hours post-transfection. For migration analysis, cells were seeded into the upper chamber of Transwell inserts (Corning, Shanghai, China) with 200 μL of serum-free medium. For invasion analysis, the upper chamber was first coated with 100 μL of Matrigel (BD, USA) that had been thawed overnight at 4°C and diluted in cold serum-free medium. After polymerization, 500 μL of 10% FBS-containing DMEM was added to the lower chamber as a chemoattractant, and 2 × 10⁵ cells in 200 μL of serum-free medium were seeded into the upper chamber. Both migration and invasion chambers were incubated at 37°C with 5% CO₂ for 48 hours. Subsequently, non-migratory/non-invasive cells on the upper surface of the membrane were gently scraped off. The remaining cells on the polycarbonate membrane were fixed with 4% paraformaldehyde for 30 minutes, permeabilized with 0.1% Triton X-100 (optional, if needed for better staining), and stained with 0.1% crystal violet in acetic acid for 20 minutes. After thorough rinsing with Phosphate-Buffered Saline (PBS) to remove excess stain, the number of migrated/invasive cells was quantified by counting three randomly selected fields per membrane under a light microscope, with the absorbance (OD) at 550 nm measured using a microplate reader. All experiments were repeated three times independently.

### 2.8 Dual-Luciferase Reporter Assay

The Dual-Luciferase Reporter Assay System (Promega) was used to assess luciferase activity in 293T cells (HonorGene, Changsha, China) co-transfected with miR-124-3p mimic or negative control (NC mimic) and wild-type (NAMPT-WT) or mutant (NAMPT-MUT) 3’UTR reporter plasmids (Sangon, Shanghai, China). Cells were seeded in 24-well plates, transfected with Lipofectamine 2000 (Invitrogen) according to the manufacturer’s protocol, and incubated for 48 hours. Firefly and Renilla luciferase activities were sequentially measured using the Dual-Glo Luciferase Assay System (Promega), with firefly activity normalized to Renilla to account for transfection efficiency. Mutant plasmids served as specificity controls, and experiments were performed in triplicate.

### 2.9 Statistical Analysis

Data are presented as mean ± Standard Deviation (SD) from at least three independent experiments. Statistical comparisons were performed using Student’s t-test for pairwise comparisons or one-way ANOVA followed by Tukey’s post hoc test for multiple group comparisons. Differences were considered statistically significant at P< 0.05. All statistical analyses were conducted using GraphPad Prism 10.0 (GraphPad Software, USA).

## 3 RESULTS

### 3.1 The Association Between NAMPT and Glioma Prognosis

To investigate the relationship between NAMPT expression and prognosis in glioma patients, we analyzed clinical data from the Chinese Glioma Genome Atlas (CGGA). Our results revealed that NAMPT expression levels were significantly associated with patient prognosis: glioma patients with high NAMPT expression exhibited markedly poorer survival outcomes compared to those with low NAMPT expression, both in primary and recurrent gliomas across all pathological grades (Fig. **1A**). Further analysis demonstrated that NAMPT expression levels increased with rising glioma grade, indicating a positive correlation between NAMPT expression and glioma pathological grade (Fig. **1B**). Additionally, NAMPT was significantly more highly expressed in IDH-wild-type gliomas than in IDH-mutant gliomas (Fig. **1C**). Collectively, these findings suggest a significant association between NAMPT expression levels and glioma prognosis.

### 3.2 Effect of miR-124-3p Overexpression on NAMPT Expression in U251 Glioma Cells

To further elucidate the functional role of miR-124-3p in glioma cell biology, we transduced U251 glioma cells with lentiviral vectors encoding either miR-124-3p mimic or a scrambled negative control (mimic-NC). Transduction efficiency was first validated using qRT-PCR. As expected, miR-124-3p expression was significantly upregulated in the mimic-transduced group compared to the mimic-NC group (Fig. **2A**). Next, we examined the impact of miR-124-3p overexpression on NAMPT at both transcriptional and translational levels. qRT-PCR analysis revealed a marked reduction in NAMPT mRNA levels following miR-124-3p mimic transduction (Fig. **2B**). Consistent with this transcriptional modulation, Western blot analysis demonstrated a significant downregulation of NAMPT protein expression in miR-124-3p mimic-treated U251 cells relative to controls (Fig. **2C**-**D**).

### 3.3 MiR-124-3p Functions as a Tumor Suppressor in Glioma by Inhibiting Cell Proliferation, Migration, Invasion, and Promoting Apoptosis

To preliminarily investigate the effects of miR-124-3p on key hallmarks of glioma progression (proliferation, invasion, and apoptosis), we employed a series of functional assays. CCK-8 for cell viability, colony formation for long-term proliferative capacity, Transwell assays for migratory/invasive potential, and flow cytometry for apoptosis analysis. Consistent with our hypothesis, miR-124-3p overexpression (via mimic transfection) exerted significant anti-tumor effects in U251 glioma cells. Specifically, the number of viable cell colonies in the miR-124-3p mimic group was significantly reduced relative to controls (Fig. **2E**-**G**). This inhibitory effect was further validated by CCK-8 assays. CCK-8 assays revealed that miR-124-3p mimic treatment markedly inhibited cell proliferation compared to the mimic-NC group after 48 hours (Fig. **2H**).

Flow cytometric apoptosis analysis demonstrated that miR-124-3p overexpression dramatically increased the apoptotic rate(LR+UR) of U251 cells (Fig. 3 A-C). Additionally, Transwell assays uncovered that miR-124-3p mimic transfection potently suppressed the invasive capability of glioma cells (Fig. **3D**-**F**). Collectively, these results establish that miR-124-3p functions as a tumor suppressor in glioma by inhibiting cell proliferation, migration, and invasion, and promoting apoptosis.

### 3.4 MiR-124-3p Negatively Regulates NAMPT Expression

NAMPT, a rate-limiting enzyme in the mammalian NAD salvage pathway, is frequently overexpressed in multiple cancer types, as extensively reported in prior studies. Bioinformatic analyses, leveraging predicted binding motifs within the 3′-untranslated region (3′-UTR) of NAMPT, have suggested that miR-124-3p may directly target this gene. To experimentally validate this interaction, we performed a dual-luciferase reporter assay. In this assay, HEK293T cells were co-transfected with either wild-type (NAMPT 3′-UTR-WT) or mutant (NAMPT 3′-UTR-MUT) luciferase constructs, engineered to contain the putative miR-124-3p binding site, or with a scrambled Negative Control (NC) oligonucleotide, alongside either miR-124-3p mimics or a non-targeting NC. Quantification of relative luciferase activity revealed no statistically significant differences between the NAMPT 3′-UTR-WT/MUT groups and their corresponding NC controls (Fig. **3G**). These results indicate that miR-124-3p does not directly modulate NAMPT expression *via* this canonical 3′-UTR interaction.

To further validate the regulatory effect of miR-124-3p on NAMPT expression, we overexpressed miR-124-3p in glioma cell lines U87 and U251. RT-qPCR and WB analyses revealed that both the transcriptional level and protein abundance of NAMPT were significantly downregulated following miR-124-3p overexpression (Fig. **4A**-**C**). These results demonstrate that miR-124-3p negatively regulates NAMPT expression. Notably, ectopic expression of an NAMPT overexpression plasmid effectively reversed the inhibitory effect of miR-124-3p overexpression on endogenous NAMPT levels (Fig. **4A**-**C**). AKT and ERK serve as key downstream regulatory targets of NAMPT. Thus, we performed WB to evaluate AKT and ERK expression levels across the three groups. Results revealed that total AKT and ERK1/2 expression remained unchanged regardless of whether miR-124-3p overexpression affected NAMPT levels (Fig. **4C**-**E**). Notably, phosphorylated AKT (p-AKT, Ser473) and phosphorylated ERK1/2(p-ERK1/2, Thr202/T185) were significantly downregulated by miR-124-3p overexpression, an effect rescued by NAMPT overexpression (Fig. **4C**-**E**). Moreover, p-AKT/p-ERK1/2 levels exhibited a positive correlation with NAMPT expression (Fig. **4C**-**E**).

### 3.5 miR-124-3p Inhibits Glioma Cell Proliferation and Viability through NAMPT Regulation

Colony formation assay demonstrated consistent results across U87 and U251 cells. MiR-124-3p overexpression significantly attenuated colony formation capacity (*p*<0.01 *vs*. Mimic NC group), whereas NAMPT overexpression effectively rescued this inhibitory effect (Fig. **5A**-**D**, *p*<0.05 *vs*. miR-124-3p mimic alone). Consistent with these findings, CCK-8 viability assays and colony formation assays revealed that miR-124-3p overexpression significantly reduced cell proliferation capacity in U251 and U87 cells compared to mimic NC controls (Fig. **5E**-**F**, *p*<0.05 at 48 hours post-transfection). Notably, this suppressive effect was effectively rescued by NAMPT overexpression (Fig. **5E**-**F**, *p*<0.05 *vs*. miR-124-3p mimic + NAMPT). These findings collectively indicate that miR-124-3p functions as a potent tumor suppressor in glioblastoma cells through its inhibitory effects on cellular proliferation, which can be mechanistically counteracted by NAMPT-mediated metabolic reprogramming.

### 3.6 miR-124-3p Regulates Glioma Cell Apoptosis by Targeting NAMPT

Elevated expression of miR-124-3p in U87 and U251 glioma cells has been demonstrated to correlate with increased apoptotic rates, prompting us to investigate its regulatory role in apoptosis *via* NAMPT modulation. To test this hypothesis, we performed flow cytometric analysis of annexin V-APC-stained U87 and U251 cells following miR-124-3p mimic transfection. As shown in Fig. (**6A**-**B**), miR-124-3p overexpression induced an 8.5-fold increase in apoptotic cells compared to the mimic NC group (*p*<0.05), characterized by annexin V-positive populations in late apoptosis (UR: 15.39% *vs*. 0.91%) in U251 cells. Strikingly, this pro-apoptotic effect was significantly attenuated by NAMPT overexpression (LR+UR: 10.10% total apoptosis, *p*<0.05). As demonstrated in Fig. (**6C**-**D**), miR-124-3p overexpression induced a 3.8-fold increase in apoptotic populations compared to mimic NC controls (*p*<0.05) in U87 glioblastoma cells. This apoptotic induction manifested as significant elevations in both early apoptosis (LR: 9.27% vs. 2.77%) and late apoptosis (UR: 9.21% *vs*. 2.04%) compartments. Strikingly, co-expression of NAMPT (pcDNA3.1-NAMPT plasmid) effectively rescued this pro-apoptotic phenotype, restoring total apoptosis rates to 8.57% (LR+UR).

### 3.7 miR-124-3p Inhibits Glioma Cell Invasion, Reversed by NAMPT Overexpression

Our transwell invasion assays revealed a significant suppression of glioblastoma cell invasion by miR-124-3p overexpression in both U251 and U87 models. As demonstrated in Fig. (**6E**-**F**), miR-124-3p mimic-transfected U251 cells exhibited a 230.8±7.98 reduction in invasive cell numbers compared to mimic NC controls (*p*<0.05), with similar inhibitory effects observed in U87 cells (Fig. **6G**-**H**, 548.1±7.432 reduction, *vs*. mimic NC, p<0.05). Notably, co-expression of NAMPT (pcDNA3.1-NAMPT plasmid) effectively rescued this invasive phenotype (Fig. **6G**-**H**, *vs*. miR-124-3p mimic, *p*<0.05).

### 3.8 STAT3 Mediates miR-124-3p Regulation of NAMPT

Previously conducted dual-luciferase reporter assays demonstrated that miR-124-3p does not directly regulate NAMPT expression through canonical binding to the 3’-UTR. The experiment provided no evidence of miR-124-3p directly targeting NAMPT, suggesting the presence of a mediating factor in this indirect regulatory axis that remains to be identified. Based on an extensive literature review and our preliminary experiments, we hypothesize that STAT3 is directly targeted by miR-124-3p, which in turn regulates the downstream expression of NAMPT. To validate this hypothesis, we performed WB analysis and rescue experiments.

Western blot analysis revealed that in the U251 glioma cell line, overexpression of miR-124-3p simultaneously supp- ressed the expression of STAT3 and NAMPT, along with inhibiting STAT3 phosphorylation (p-STAT3, Tyr705), suggesting that miR-124-3p inversely regulates STAT3 and NAMPT expression (Fig. **7A**-**B**). Following STAT3 knockdown, NAMPT expression was significantly reduced in U251 cells. Conversely, overexpression of STAT3 reversed the miR-124-3p overexpression-induced suppression of NAMPT expression, indicating that NAMPT is regulated by upstream STAT3 (Fig. **7A**-**B**). Since the direct binding of miR-124-3p to the 3'-UTR of STAT3 mRNA has been well-established by dual-luciferase reporter assays [20], STAT3 is thus identified as an intermediate regulator that is directly modulated by miR-124-3p and subsequently controls the expression of downstream NAMPT.

In addition, we examined another apoptosis-related indicator, the expression ratio of Bcl-2/Bax. In the miR-124-3p mimic group, the expression of BAX was significantly increased, while the expression of BCL-2 was markedly downregulated, leading to a substantial reduction in the Bcl-2/Bax ratio (Fig. **7A**-**B**). These findings suggest that overexpression of miR-124-3p significantly promotes apoptosis in glioma cells. Importantly, overexpression of STAT3 was able to reverse the miR-124-3p-induced decrease in the Bcl-2/Bax ratio. These results are consistent with our previous flow cytometry data, collectively demonstrating that miR-124-3p can significantly enhance glioma cell apoptosis.

## 4 DISCUSSION

Our preliminary studies identified 59 differentially expressed microRNAs (miRNAs) associated with malignant biological behaviors in gliomas, with miR-124-3p emerging as a candidate of particular interest. This observation aligns with multiple bioinformatics analyses indicating that miR-124-3p exhibits regulatory functions across diverse cancer types [21-24]. Accumulating evidence demonstrates that miR-124-3p functions as a potent tumor suppressor across diverse malignancies through distinct molecular mechanisms. In gastric cancer, it inhibits tumorigenesis by directly targeting integrin β1 (ITGB1) [25]. Conversely, reduced miR-124-3p expression correlates with breast cancer progression and metastasis via modulation of MGAT5-mediated N-glycosylation pathways [26]. Notably, this miRNA suppresses metastatic dissemination in Non-Small Cell Lung Cancer (NSCLC) through dual mechanisms: regulating extracellular exosome trafficking and modulating intracellular PI3K/AKT signaling cascades [27]. In bladder cancer, miR-124-3p governs cell proliferation-apoptosis balance by repressing endothelin receptor type B (EDNRB) expression, thereby influencing tumor growth dynamics [28]. These studies collectively highlight its conserved tumor-suppressive role while revealing context-dependent regulatory patterns across cancer types. Collectively, these findings demonstrate that while miR-124-3p engages in context-dependent molecular mechanisms to regulate tumor biology, it consistently functions as a potent tumor suppressor by inhibiting key oncogenic processes, including tumor cell proliferation, migration, and invasion, across diverse malignancies. Furthermore, miR-124-3p exhibits significant differential expression between normal tissues and tumor cells in multiple cancer types, a characteristic that positions it as a promising diagnostic and prognostic biomarker [13, 29, 30].

Nicotinamide Adenine Dinucleotide (NAD+) serves as a central metabolic and redox cofactor critical for maintaining cellular bioenergetic homeostasis and regulating longevity pathways [31]. This pyridine nucleotide participates in redox reactions through reversible interconversion with its reduced form, NADH, functioning as an indispensable cofactor in multiple enzymatic systems, including glycolysis, oxidative phosphorylation, fatty acid β-oxidation, and the tricarboxylic acid (TCA) cycle [32]. Emerging evidence demonstrates that NAD+ metabolism undergoes profound dysregulation during oncogenesis. Tumor cells exhibit altered NAD+ biosynthesis pathways through upregulated salvage pathways involving NAMPT, the rate-limiting enzyme catalyzing NAD+ regeneration from nicotinamide [33-34].

Accumulating evidence from multiple independent studies has consistently demonstrated elevated NAMPT expression in glioma tissues, a finding corroborated by our current investigations. Notably, this upregulation exhibits a pathological grade-dependent pattern, with higher NAMPT levels correlating with advanced glioma grades [35]. Retrospective analysis of clinical cohorts from the CGGA and TCGA databases further reveals an inverse relationship between NAMPT expression levels and patient survival outcomes, establishing NAMPT as a robust prognostic biomarker in gliomas [36]. Emerging mechanistic insights implicate NAMPT in modulating glioma stemness characteristics.

Previous studies have consistently demonstrated significantly elevated expression of NAMPT in glioma tissues compared to normal brain tissues, a finding that aligns with our current investigation [35]. Furthermore, through comprehensive analysis of the CGGA cohort, we identified an inverse relationship between NAMPT expression and patient prognosis, where higher NAMPT levels correlated with poorer survival outcomes (*p*<0.05). This prognostic significance was further validated in an independent TCGA glioblastoma dataset, which demonstrated consistent trends in survival analysis (*p*< 0.01) [36, 37]. These converging lines of evidence from multiple genomic databases underscore NAMPT's critical role in glioma progression and therapeutic resistance.

Emerging evidence indicates that NAMPT exerts context-dependent regulatory effects on glioma malignant progression through distinct molecular mechanisms. Notably, accumulating studies implicate NAMPT in glioma stemness regulation, where its ectopic overexpression in glioma cell lines significantly enhances tumorigenic potential by modulating embryonic stem cell pathways and expanding the cancer-initiating cell population [37-39]. Preclinical investigations have established the robust anticancer efficacy of NAMPT inhibitors, particularly in IDH1/2-mutant cancer cell populations. Notably, patient-derived primary glioblastoma cell lines harboring IDH1/2 mutations, including MGG119, MGG152, and BT142, demonstrate heightened sensitivity to NAMPT-targeted pharmacological inhibition [40].

Based on our preliminary bioinformatics analysis, we hypothesized that NAMPT is directly regulated by miR-124-3p. However, dual-luciferase reporter assays revealed that miR-124-3p does not directly modulate NAMPT expression through canonical binding to its 3'-untranslated region (3'-UTR). This discrepancy led us to propose the existence of an intermediate regulatory factor between miR-124-3p and NAMPT.

Emerging evidence indicates that miR-124-3p exerts significant negative regulation over STAT3 (and its phosphorylated form p-STAT3) *via* direct binding to the 3'-UTR of STAT3 mRNA, as confirmed by dual-luciferase reporter assays [20, 41, 42]. This interaction suppresses STAT3 post-transcriptional expression, leading to reduced total STAT3 protein levels and diminished p-STAT3 activity [43]. Notably, STAT3 has been shown to function as a transcription factor that directly binds to the NAMPT promoter in tumors such as lymphomas, thereby enhancing NAMPT expression to promote NAD+ metabolism and support tumor cell survival [44]. To validate this hypothetical regulatory axis, we performed Western Blot (WB) and rescue experiments. Our results demonstrated that miR-124-3p-mediated suppression of STAT3 expression inversely correlates with NAMPT protein levels, confirming that STAT3 acts as an intermediate regulator in this pathway. These findings establish a novel miR-124-3p-STAT3-NAMPT regulatory cascade that modulates NAD+ metabolism in tumor biology.

Extracellular NAMPT secreted by melanoma cells and NAMPT overexpression in breast cancer cells have both been associated with AKT activation [45, 46]. Notably, exogenous eNAMPT promotes breast cancer cell proliferation by inducing AKT and ERK1/2 phosphorylation, a process that can be blocked by AKT or ERK1/2 inhibitors [47]. In multiple cancer models, NAMPT inhibition has been shown to reduce ERK1/2 phosphorylation, and combined treatment with NAMPT inhibitors and ERK1/2 blockers synergistically enhances apoptosis [48-50]. Our experimental results demonstrate that reduced NAMPT expression induced by miR-124-3p overexpression does not affect total AKT or ERK1/2 protein levels; however, it significantly diminishes their phosphorylation. Rescue experiments further revealed that restoring NAMPT expression reverses the reduction in AKT and ERK phosphorylation.

AKT and ERK serve as critical effector molecules in the PI3K/AKT and MEK/ERK signaling transduction pathways, respectively [51, 52]. The biological activity of AKT and ERK depends entirely on their phosphorylation status: unphosphorylated forms remain inactive, unable to bind downstream substrates or transmit signaling cascades. Notably, these two pathways exhibit intricate cross-talk, with their phosphorylation dynamics exerting synergistic effects on tumorigenesis through context-dependent mechanisms [53]. Collectively, these observations suggest that the miR-124-3p/NAMPT regulatory axis suppresses both the MEK/ERK and PI3K/AKT signaling pathways.

Our study is subject to certain limitations, which should be acknowledged. All experimental data were derived from *in vitro* cellular assays, and as such, these findings have not undergone *in vivo* validation. In future work, we aim to address this gap by validating the tumor-suppressive effects of miR-124-3p, alongside previously validated miRNAs, in *in vivo* models. We will also conduct further mechanistic investigations to clarify the underlying pathways involved.

## CONCLUSION

Our study demonstrates that miR-124-3p is significantly downregulated in glioma tissues, and its expression levels correlate closely with patient prognosis and survival outcomes. These findings underscore the potential of miR-124-3p as a clinically valuable biomarker for both diagnostic and therapeutic applications. MiR-124-3p directly targets and suppresses STAT3 expression, thereby indirectly regulating NAMPT expression. As the rate-limiting enzyme catalyzing NAD+ regeneration from nicotinamide, NAMPT modulates downstream ERK and AKT signaling pathways, ultimately influencing the malignant biological behavior of glioma cells.

## Figures and Tables

**Fig. (1):**
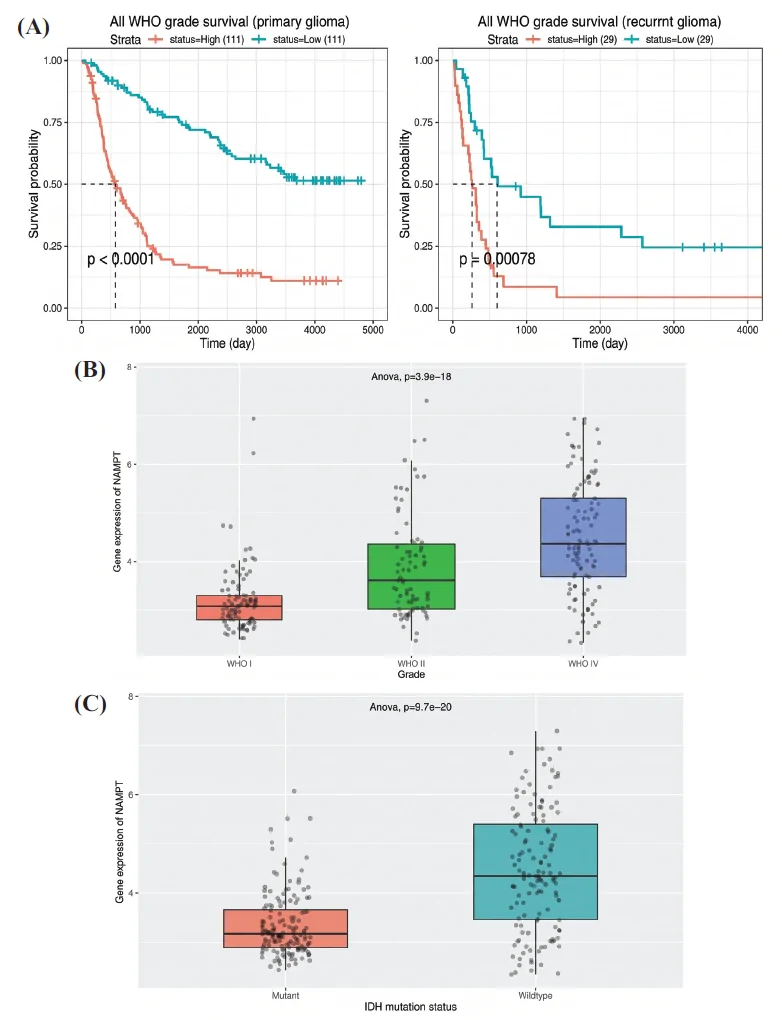
Association of NAMPT expression with prognosis, tumor grade, and IDH mutation status in gliomas: A CGGA database analysis. (**A**) In all patients with primary and recurrent gliomas, NAMPT expression was negatively correlated with prognosis, with higher NAMPT expression levels associated with poorer clinical outcomes. (**B**) NAMPT expression was positively correlated with pathological grade in gliomas. (**C**) NAMPT expression was significantly higher in IDH-wild-type gliomas compared to IDH-mutant gliomas.

**Fig. (2):**
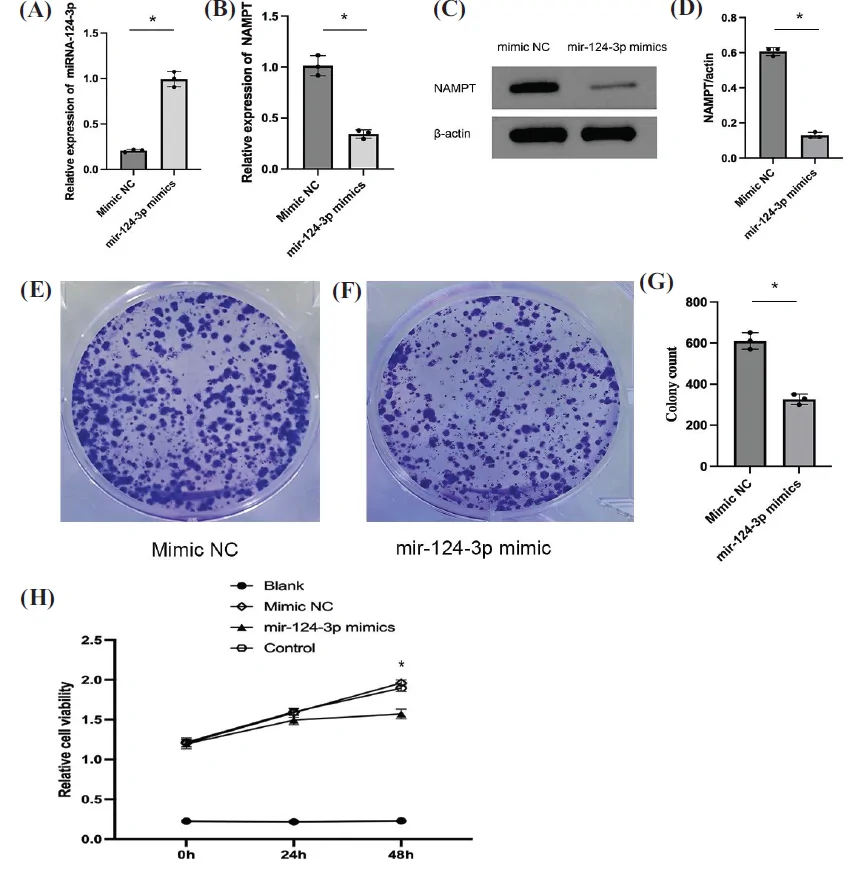
Overexpression of miR-124-3p suppresses NAMPT protein expression, reduces glioma cell viability, and inhibits cellular proliferation. (**A**) RT-PCR showed that miR-124-3p expression was significantly upregulated in U251 cells following transfection. (**B**) Following transfection with miR-124-3p, PCR demonstrated significantly reduced NAMPT expression in U251 cells. (**C**-**D**) WB showed significantly reduced NAMPT protein expression in U251 cells following transfection with miR-124-3p. (**E**-**G**) Colony formation assay demonstrated that cell proliferation was significantly reduced in U251 cells following miR-124-3p transfection compared to the control group. (**H**) CCK-8 assay showed that U251 cell viability at 48 hours post-transfection was significantly lower than that in the untransfected (mimic NC) group and the control group. Each experiment was repeated at least three times. ^∗^*p*< 0:05.

**Fig. (3):**
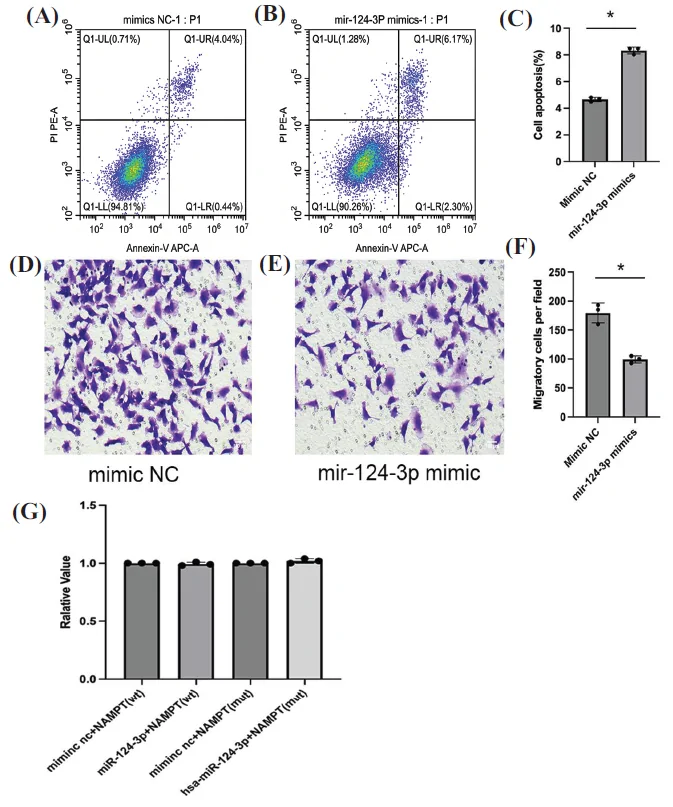
Overexpression of miR-124-3p promotes apoptosis and inhibits proliferation in glioma cells. (**A**-**C**) Flow cytometric apoptosis assay demonstrated that the total apoptotic rate of U251 cells transfected with miR-124-3p was significantly higher than that in the NC group after 48h(LR: early apoptosis; UR: late apoptosis; total apoptotic rate: LR + UR). (**D**-**F**) The Transwell migration assay showed that the number of migrated cells was significantly reduced following transfection with miR-124-3p. Fig. (**3g**) Quantification of relative luciferase activity revealed no statistically significant differences between the NAMPT 3′-UTR-WT/MUT groups and their corresponding NC controls. Each experiment was repeated at least three times. ^∗^*p*< 0:05.

**Fig. (4):**
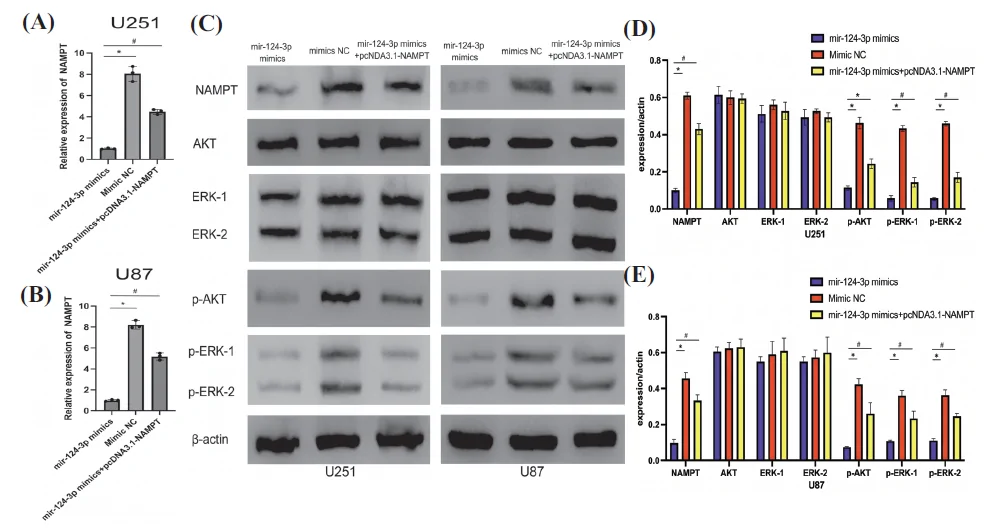
The overexpression of miR-124-3p affects the expression levels of NAMPT, AKT, and ERK1/2. (**A**-**B**) RT-PCR demonstrated that the NAMPT overexpression plasmid reversed the miR-124-3p overexpression-induced reduction in NAMPT transcriptional level in U251 and U87 glioma cell lines. (**C**-**E**) Western blotting analysis revealed that in U251 and U87 glioma cell lines, overexpression of miR-124-3p mimic significantly reduced NAMPT protein levels, an effect that was rescued by NAMPT overexpression plasmid through restoring NAMPT expression; additionally, neither miR-124-3p mimic nor NAMPT overexpression exerted notable impacts on the total protein levels of downstream AKT or ERK1/2, whereas in the miR-124-3p mimic group, phosphorylation levels of AKT and ERK1/2 were markedly suppressed, and this inhibitory effect was reversed by NAMPT overexpression. Each experiment was repeated at least three times. Mir-124-3p mimic vs. Mimic NC, ^∗^P< 0:05; Mir-124-3p mimic vs. Mir-124-3p mimic + pcNDA3.1-gene, ^#^*p*< 0:05.

**Fig. (5):**
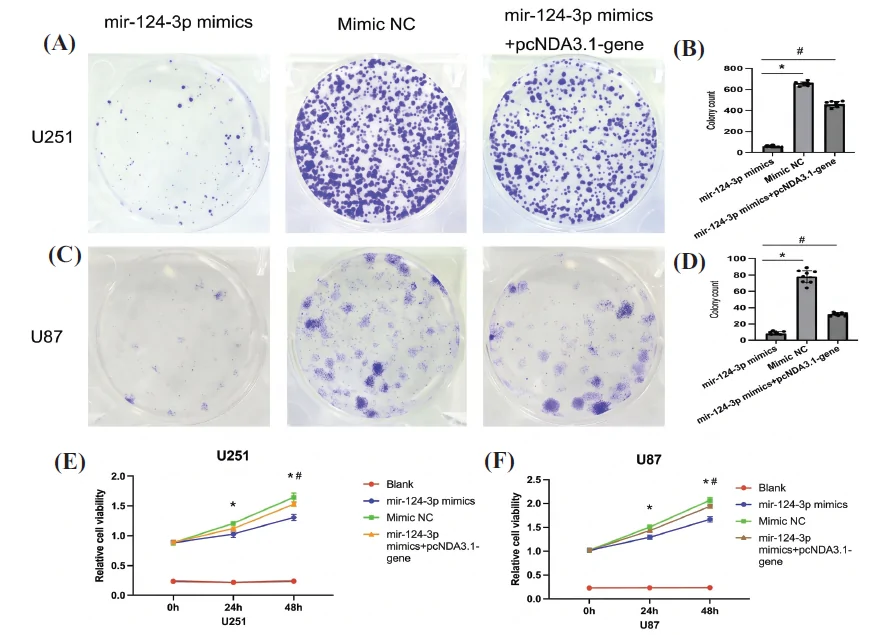
The overexpression of miR-124-3p inhibits the viability and proliferation of glioma cells, while NAMPT rescues this effect. (**A**-**D**) Colony formation assay demonstrated that in U251 and U87 cell lines, overexpression of miR-124-3p inhibited cell proliferation, while overexpression of NAMPT rescued this inhibitory effect. (**E**, **F**) CCK-8 assay demonstrated that after 48 hours of incubation in U251 and U87 cell lines, overexpression of miR-124-3p inhibited cell viability, while overexpression of NAMPT rescued this inhibitory effect. Each experiment was repeated at least three times. Mir-124-3p mimic vs. Mimic NC, ^∗^*p*< 0:05; Mir-124-3p mimic *vs*. Mir-124-3p mimic + pcNDA3.1-gene, ^#^*p*< 0:05.

**Fig. (6):**
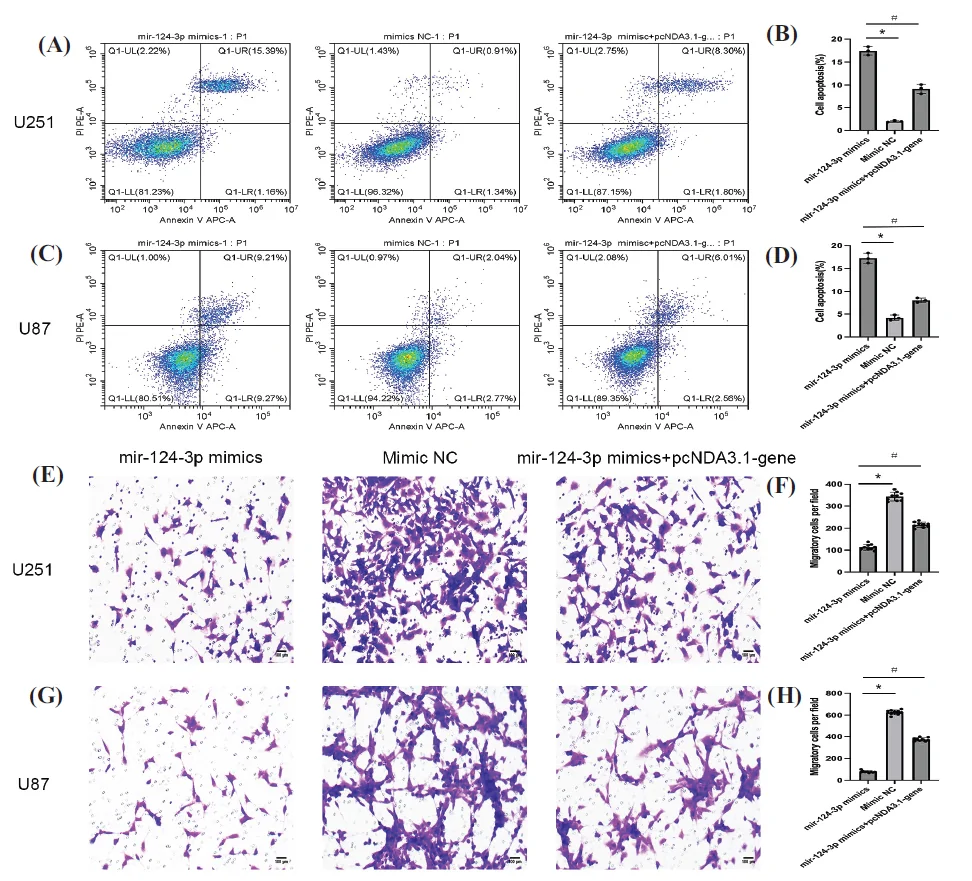
The overexpression of miR-124-3p promotes apoptosis and inhibits the invasion of glioma cells, while NAMPT rescues these effects. Flow cytometric apoptosis assay revealed that after 48 hours of incubation. (**A**, **B**) In U251 cells, the total apoptotic rate in the miR-124-3p mimic group was significantly higher than that in the NC group, and this apoptotic increase was reversed by NAMPT overexpression. (**C**, **D**) In U87 cells, both the early apoptotic rate and total apoptotic rate in the miR-124-3p mimic group were significantly elevated compared to the NC group, and this enhancement was similarly reversed by NAMPT overexpression. (**E**, **H**) Transwell assay demonstrated that in U251 and U87 cells, cell invasion was significantly inhibited in the miR-124-3p mimic group, and this inhibitory effect was rescued by NAMPT overexpression. Each experiment was repeated at least three times. Mir-124-3p mimic *vs*. Mimic NC, ^∗^P< 0:05; Mir-124-3p mimic *vs*. Mir-124-3p mimic + pcNDA3.1-gene, ^#^*p*< 0:05.

**Fig. (7):**
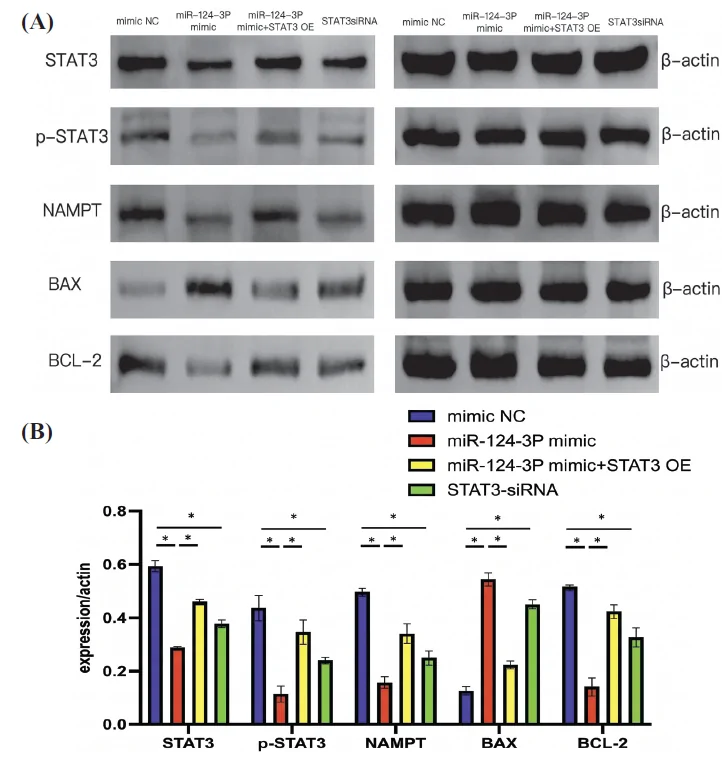
miR-124-3p directly regulates STAT3, which in turn modulates the expression of its downstream target NAMPT. (**A**, **B**) Western blotting analysis revealed that in U251 glioma cell lines, in the miR-124-3p mimic group, both STAT3 expression and its phosphorylated form (p-STAT3) were significantly downregulated. Overexpression of STAT3 reversed the inhibitory effects of miR-124-3p mimic on STAT3 and p-STAT3 expression. Furthermore, STAT3 overexpression restored NAMPT expression that had been suppressed by miR-124-3p overexpression. Notably, specific knockdown of STAT3 alone significantly reduced NAMPT expression levels in U251 cells, indicating that NAMPT is regulated by upstream STAT3 expression. Overexpression of miR-124-3p significantly increased Bax protein expression while markedly decreasing BCL-2 expression. Co-overexpression of both miR-124-3p and STAT3 rescued these changes. Furthermore, STAT3 knockdown alone was sufficient to induce similar alterations in Bax and BCL-2 protein levels. Each experiment was repeated at least three times. ^∗^*p*< 0:05.

**Table 1:** RT-PCR primer sequences.

**H-actin**	**F: ACCCTGAAGTACCCCATCGAG**
-	R: AGCACAGCCTGGATAGCAAC
H-NAMPT	F: ATCCTGTTCCAGGCTATTCTGT
-	R: CCCCATATTTTCTCACACGCAT
hsa-miR-124-3p	TAAGGCACGCGGTGAATGCCAA

## Data Availability

The data and supportive information are available within the article.

## LIST OF ABBREVIATIONS

CGGA: Chinese Glioma Genome Atlas
miRNAs: MicroRNAs
HR: Higher Hazard Ratio
IDH: Isocitrate Dehydrogenase
HGG: High-grade Gliomas
NAD: Nicotinamide Adenine Dinucleotide

## References

[1] Zafar S., Hafeez A., Shah H., Mutiullah I., Ali A., Khan K., Figueroa-González G., Reyes-Hernández O.D., Quintas-Granados L.I., Peña-Corona S.I., Kiyekbayeva L.N., Butnariu M., Tota C.E., Caunii A., Büsselberg D., Sharifi-Rad J., Leyva-Gómez G. (2025). Emerging biomarkers for early cancer detection and diagnosis: Challenges, innovations, and clinical perspectives.. Eur J. Med. Res..

[2] Gupta A., Mehrotra A., Sood A., Sharma B., Yadav V., Kaur G., Sak K., Satapathy S.R., Tuli H.S. (2025). MicroRNAs at the crossroad of cancer therapeutics: Insights from WNT signaling & flavonoids.. Front. Mol. Biosci.

[3] Pourrahimi M., Hesari M., Houshmandpour H., Mirzaee O., Fouladseresht H., Torki E., Kouchaki H., Tabrizi R., Ghasemian A., Barbaresi S. (2025). Inducer microRNAs in the glioma development: A concise review of mechanisms and insights into targeted therapy.. J. Egypt Natl. Canc Inst.

[4] Hartanto R.A., Malueka R.G., Tamba D.A., Lukito P.P., Silvano F., Sutarni S. (2025). The role of miRNA-10b and miRNA-21 in radioresistance and temozolomide resistance of high-grade glioma patients: A systematic review.. Neurosurg. Focus.

[5] Wang S., Yin Y., Liu S. (2019). Roles of microRNAs during glioma tumorigenesis and progression.. Histol Histopathol.

[6] Ghaemmaghami A.B., Mahjoubin-Tehran M., Movahedpour A., Morshedi K., Sheida A., Taghavi S.P., Mirzaei H., Hamblin M.R. (2020). Role of exosomes in malignant glioma: MicroRNAs and proteins in pathogenesis and diagnosis.. Cell. Commun. Signal..

[7] Wang L., Liu Y., Yu Z., Gong J., Deng Z., Ren N., Zhong Z., Cai H., Tang Z., Cheng H., Chen S., He Z. (2021). Mir-139-5p inhibits glioma cell proliferation and progression by targeting GABRA1.. J. Transl. Med..

[8] Yu Z., Liu Y., Li Y., Zhang J., Peng J., Gong J., Xia Y., Wang L. (2022). miRNA-338-3p inhibits glioma cell proliferation and progression by targeting MYT1L.. Brain Res. Bull.

[9] Gong J., Tang Z., Yu Z., Deng Z., Liu Y., Ren N., Wang L., He Z. (2022). miR-138-5p inhibits the growth and invasion of glioma cells by regulating WEE1.. Anal. Cell. Pathol..

[10] Yu Z., Li J., Jiang J., Li Y., Lin L., Xia Y., Wang L. (2023). miRNA-128-3p inhibits malignant behavior of glioma cells by downregulating KLHDC8A expression.. Nan Fang Yi Ke Da Xue Xue Bao.

[11] Liu Y., Wang Z., Tang Z., Fu Y., Wang L. (2024). mirna-383-5p functions as an anti-oncogene in glioma through the Akt/mTOR signaling pathway by targeting VEGFA.. Curr. Cancer Drug Targets.

[12] Aman R.A., Pratama M.G., Satriawan R.R., Ardiansyah I.R., Suanjaya I.K.A. (2024). Diagnostic and prognostic values of mirnas in high-grade gliomas: A systematic review.. F1000 Res..

[13] Olioso D., Caccese M., Santangelo A., Lippi G., Zagonel V., Cabrini G., Lombardi G., Dechecchi M.C. (2021). Serum exosomal microRNA-21, 222 and 124-3p as noninvasive predictive biomarkers in newly diagnosed high-grade gliomas: A prospective study.. Cancers.

[14] Clausing M., William D., Preussler M., Biedermann J., Grützmann K., Richter S., Buchholz F., Temme A., Schröck E., Klink B. (2022). Different effects of RNAi-Mediated downregulation or chemical inhibition of NAMPT in an Isogenic IDH mutant and wild-type glioma cell model.. Int. J. Mol. Sci..

[15] Bombino A., Magnani M., Conti A. (2024). A promising breakthrough: The potential of VORASIDENIB in the treatment of low-grade glioma.. Curr. Mol. Pharmacol..

[16] Liang F., You Q., Yu B., Wang C., Yang Y., Zhu L., He Z. (2025). Neurotransmitter‐mimicking nanovesicles facilitate postoperative glioblastoma stem cell‐specific treatment for preventing tumor recurrence.. Adv. Sci..

[17] Mellinghoff I.K., van den Bent M.J., Blumenthal D.T., Touat M., Peters K.B., Clarke J., Mendez J., Yust-Katz S., Welsh L., Mason W.P., Ducray F., Umemura Y., Nabors B., Holdhoff M., Hottinger A.F., Arakawa Y., Sepulveda J.M., Wick W., Soffietti R., Perry J.R., Giglio P., de la Fuente M., Maher E.A., Schoenfeld S., Zhao D., Pandya S.S., Steelman L., Hassan I., Wen P.Y., Cloughesy T.F. (2023). Vorasidenib in IDH1- or IDH2-Mutant low-grade glioma.. N Engl. J. Med..

[18] Sampath D., Zabka T.S., Misner D.L., O’Brien T., Dragovich P.S. (2015). Inhibition of nicotinamide phosphoribosyltransferase (NAMPT) as a therapeutic strategy in cancer.. Pharmacol. Ther..

[19] Lin T.C. (2022). Updated functional roles of NAMPT in carcinogenesis and therapeutic niches.. Cancers.

[20] Wang S., Wu G., Han Y., Song P., Chen J., Wu Y., Yang J., Liang P. (2018). miR-124 regulates STAT3-mediated cell proliferation, migration and apoptosis in bladder cancer.. Oncol. Lett..

[21] Romero-López M.J., Jiménez-Wences H., Cruz-De la Rosa M.I., Román-Fernández I.V., Fernández-Tilapa G. (2022). miR-23b-3p, miR-124-3p and miR-218-5p synergistic or additive effects on cellular processes that modulate cervical cancer progression? A molecular balance that needs attention.. Int. J. Mol. Sci..

[22] Li Q., Liu S., Yan J., Sun M.Z., Greenaway F.T. (2021). The potential role of miR-124-3p in tumorigenesis and other related diseases.. Mol. Biol. Rep..

[23] Zottel A., Šamec N., Kump A., Raspor Dall’Olio L.R., Pužar Dominkuš P., Romih R., Hudoklin S., Mlakar J., Nikitin D., Sorokin M., Buzdin A., Jovčevska I., Komel R. (2020). Analysis of miR-9-5p, miR-124-3p, miR-21-5p, miR-138-5p, and miR-1-3p in glioblastoma cell lines and extracellular vesicles.. Int. J. Mol. Sci..

[24] Romero-López M.J., Jiménez-Wences H., Cruz-De La Rosa M.I., Alarcón-Millán J., Mendoza-Catalán M.Á., Ortiz-Sánchez E., Tinajero-Rodríguez J.M., Hernández-Sotelo D., Valente-Niño G.W., Martínez-Carrillo D.N., Fernández-Tilapa G. (2024). miR-218-5p, miR-124-3p and miR-23b-3p act synergistically to modulate the expression of NACC1, proliferation, and apoptosis in C-33A and CaSki cells.. Noncoding RNA Res..

[25] Li Q., Zhou L., Liang Q., Qin H., Chen Y., Wang T., Wei Q., Luo Y., Li G., Huang H. (2025). MiR-124-3p inhibits proliferation, migration, and epithelial-mesenchymal transformation in gastric cancer by targeting ITGB1.. Am J. Transl. Res..

[26] Yan G., Li Y., Zhan L., Sun S., Yuan J., Wang T., Yin Y., Dai Z., Zhu Y., Jiang Z., Liu L., Fan Y., Yang F., Hu W. (2019). Decreased miR-124-3p promoted breast cancer proliferation and metastasis by targeting MGAT5.. Am J. Cancer Res..

[27] Zhu Q., Zhang Y., Li M., Zhang Y., Zhang H., Chen J., Liu Z., Yuan P., Yang Z., Wang X. (2023). MiR-124-3p impedes the metastasis of non-small cell lung cancer via extracellular exosome transport and intracellular PI3K/AKT signaling.. Biomark. Res..

[28] Fu W., Wu X., Yang Z., Mi H. (2019). The effect of miR-124-3p on cell proliferation and apoptosis in bladder cancer by targeting EDNRB.. Arch. Med. Sci..

[29] Wang C., Fu R., Wang Y., Wei J., Yu Y., Hu L., Zhang C. (2024). miR-124-3p and miR-194-5p regulation of the PI3K/AKT pathway via ROR2 in medulloblastoma progression.. Cancer Gene Ther..

[30] Jin L., Zhang Z. (2020). Serum miR-3180-3p and miR-124-3p may function as noninvasive biomarkers of cisplatin resistance in gastric cancer.. Clin. Lab..

[31] Navas L.E., Carnero A. (2021). NAD+ metabolism, stemness, the immune response, and cancer.. Signal. Transduct. Target Ther..

[32] Xie N., Zhang L., Gao W., Huang C., Huber P.E., Zhou X., Li C., Shen G., Zou B. (2020). NAD+ metabolism: Pathophysiologic mechanisms and therapeutic potential.. Signal. Transduct. Target Ther..

[33] Indini A., Fiorilla I., Ponzone L., Calautti E., Audrito V. (2022). NAD/NAMPT and mTOR pathways in melanoma: Drivers of drug resistance and prospective therapeutic targets.. Int. J. Mol. Sci..

[34] Zhang W., Ren H., Chen W., Hu B., Feng C., Li P., Shi Y., Fang J. (2025). Nicotinamide phosphoribosyltransferase in NAD+ metabolism: Physiological and pathophysiological implications.. Cell. Death Discov..

[35] Vachher M., Arora K., Burman A., Kumar B. (2020). NAMPT, GRN, and SERPINE1 signature as predictor of disease progression and survival in gliomas.. J. Cell. Biochem..

[36] Guo Q., Han N., Shi L., Yang L., Zhang X., Zhou Y., Yu S., Zhang M. (2019). NAMPT: A potential prognostic and therapeutic biomarker in patients with glioblastoma.. Oncol. Rep..

[37] Gujar A.D., Le S., Mao D.D., Dadey D.Y.A., Turski A., Sasaki Y., Aum D., Luo J., Dahiya S., Yuan L., Rich K.M., Milbrandt J., Hallahan D.E., Yano H., Tran D.D., Kim A.H. (2016). An NAD + -dependent transcriptional program governs self-renewal and radiation resistance in glioblastoma.. Proc. Natl. Acad. Sci. USA.

[38] Lucena-Cacace A., Otero-Albiol D., Jiménez-García M.P., Peinado-Serrano J., Carnero A. (2017). NAMPT overexpression induces cancer stemness and defines a novel tumor signature for glioma prognosis.. Oncotarget.

[39] Lucena-Cacace A., Umeda M., Navas L.E., Carnero A. (2019). NAMPT as a dedifferentiation-inducer gene: NAD+ as core axis for glioma cancer stem-like cells maintenance.. Front. Oncol..

[40] Tateishi K., Wakimoto H., Iafrate A.J., Tanaka S., Loebel F., Lelic N., Wiederschain D., Bedel O., Deng G., Zhang B., He T., Shi X., Gerszten R.E., Zhang Y., Yeh J.R.J., Curry W.T., Zhao D., Sundaram S., Nigim F., Koerner M.V.A., Ho Q., Fisher D.E., Roider E.M., Kemeny L.V., Samuels Y., Flaherty K.T., Batchelor T.T., Chi A.S., Cahill D.P. (2015). Extreme vulnerability of IDH1 mutant cancers to NAD+ depletion.. Cancer Cell..

[41] Geng L., Liu W., Chen Y. (2017). miR-124-3p attenuates MPP + -induced neuronal injury by targeting STAT3 in SH-SY5Y cells.. Exp. Biol. Med..

[42] Chen Z., Liu Y., Wu X., Lin W., Liu Z., Huang Y., Chen Y., Tang Y., Chen A., Lin C. (2023). Spinal CircKcnk9 regulates chronic visceral hypersensitivity of irritable bowel syndrome.. J. Pain.

[43] Liu S., Gong J. (2022). miR‐124‐3p delivered using exosomes attenuates the keratinocyte response to IL‐17A stimulation in psoriasis.. Oxid Med. Cell. Longev.

[44] Zhang Q., Basappa J., Wang H.Y., Nunez-Cruz S., Lobello C., Wang S., Liu X., Chekol S., Guo L., Ziober A., Nejati R., Shestov A., Feldman M., Glickson J.D., Turner S.D., Blair I.A., Van Dang C., Wasik M.A. (2023). Chimeric kinase ALK induces expression of NAMPT and selectively depends on this metabolic enzyme to sustain its own oncogenic function.. Leukemia.

[45] Grolla A.A., Torretta S., Gnemmi I., Amoruso A., Orsomando G., Gatti M., Caldarelli A., Lim D., Penengo L., Brunelleschi S., Genazzani A.A., Travelli C. (2015). Nicotinamide phosphoribosyltransferase ( NAMPT / PBEF /visfatin) is a tumoural cytokine released from melanoma.. Pigment Cell. Melanoma Res..

[46] Ge X., Zhao Y., Dong L., Seng J., Zhang X., Dou D. (2019). NAMPT regulates PKM2 nuclear location through 14‐3‐3ζ: Conferring resistance to tamoxifen in breast cancer.. J. Cell. Physiol..

[47] Wei Y., Xiang H., Zhang W. (2022). Review of various NAMPT inhibitors for the treatment of cancer.. Front. Pharmacol..

[48] Cea M., Cagnetta A., Fulciniti M., Tai Y.T., Hideshima T., Chauhan D., Roccaro A., Sacco A., Calimeri T., Cottini F., Jakubikova J., Kong S.Y., Patrone F., Nencioni A., Gobbi M., Richardson P., Munshi N., Anderson K.C. (2012). Targeting NAD+ salvage pathway induces autophagy in multiple myeloma cells via mTORC1 and extracellular signal-regulated kinase (ERK1/2) inhibition.. Blood.

[49] Okumura S., Sasaki T., Minami Y., Ohsaki Y. (2012). Nicotinamide phosphoribosyltransferase: A potent therapeutic target in non-small cell lung cancer with epidermal growth factor receptor-gene mutation.. J. Thorac Oncol..

[50] Zhang L.Y., Liu L.Y., Qie L.L., Ling K.N., Xu L.H., Wang F., Fang S.H., Lu Y.B., Hu H., Wei E.Q., Zhang W.P. (2012). Anti-proliferation effect of APO866 on C6 glioblastoma cells by inhibiting nicotinamide phosphoribosyltransferase.. Eur J. Pharmacol..

[51] Peng Y., Wang Y., Zhou C., Mei W., Zeng C. (2022). PI3K/Akt/mTOR pathway and its role in cancer therapeutics: Are we making headway?. Front. Oncol..

[52] Fontana F., Giannitti G., Marchesi S., Limonta P. (2024). The PI3K/Akt pathway and glucose metabolism: A dangerous liaison in cancer.. Int. J. Biol. Sci..

[53] Li Q., Li Z., Luo T., Shi H. (2022). Targeting the PI3K/AKT/mTOR and RAF/MEK/ERK pathways for cancer therapy.. Mol. Biomed..

